# Empirical formulation of broadband complex refractive index spectra of single-chirality carbon nanotube assembly

**DOI:** 10.1515/nanoph-2021-0728

**Published:** 2022-01-12

**Authors:** Taishi Nishihara, Akira Takakura, Masafumi Shimasaki, Kazunari Matsuda, Takeshi Tanaka, Hiromichi Kataura, Yuhei Miyauchi

**Affiliations:** Institute of Advanced Energy, Kyoto University, Uji 611-0011, Kyoto, Japan; Nanomaterials Research Institute, National Institute of Advanced Industrial Science and Technology (AIST), Tsukuba 305-8565, Ibaraki, Japan

**Keywords:** carbon nanotube, complex refractive index, exciton, thermo-optic device, thin film

## Abstract

Assemblies of single-walled carbon nanotubes with a specific chiral structure are promising future optofunctional materials because of their strong light–matter coupling arising from sharp optical resonances of quasi-one-dimensional excitons. Their strong optical resonances, which lie in the infrared-to-visible wavelength region, can be selected by their chiralities, and this selectivity promises a wide range of applications including photonic and thermo-optic devices. However, the broadband complex optical spectra of single-chirality carbon nanotube assemblies are scarce in the literature, which has prevented researchers and engineers from designing devices using them. Here, we experimentally determine broadband complex refractive index spectra of single-chirality carbon nanotube assemblies. Free-standing carbon nanotube membranes and those placed on sapphire substrates were fabricated via filtration of the nanotube solution prepared by the separation method using gel chromatography. Transmission and reflection spectra were measured in the mid-infrared to visible wavelength region, and the complex refractive indices of nanotube assemblies were determined as a function of photon energy. The real and imaginary parts of the refractive indices of the nanotube membrane with a bulk density of 1 g cm^−3^ at the first subband exciton resonance were determined to be approximately 2.7–3.6 and 1.3i–2.4i, respectively. We propose an empirical formula that phenomenologically describes the complex refractive index spectra of various single-chirality nanotube membranes, which can facilitate the design of photonic devices using carbon nanotubes as the material.

## 1 Introduction

**Figure 1:**
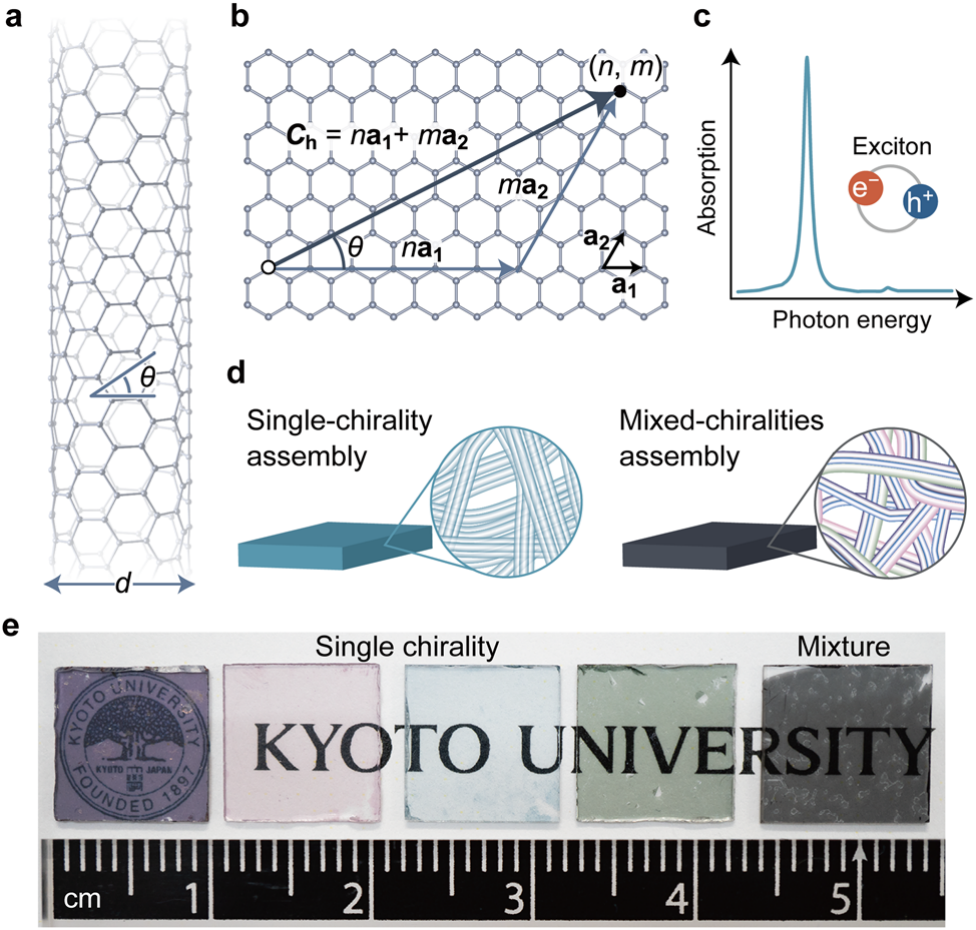
Single-chirality CNT assembly. (a) Schematic of single-walled carbon nanotube (SWCNT) with diameter *d* and chiral angle *θ*. (b) Definition of chiral indices (*n*, *m*). ***C***_h_ is a chiral vector connecting two carbon atoms in a graphene plane, and an SWCNT is formed by rolling up to connect them. (*n*, *m*) are the coordinates with respect to the bases **a**_1_ and **a**_2_. (c) Schematic of excitonic optical absorption in a quasi-one-dimensional system. (d) SWCNT assemblies of a single chirality and those of mixed chiralities. (e) Single-chirality SWCNT membranes on sapphire substrates, together with a mixed-chirality membrane. Schematics in (a) and (b) were drawn using VESTA 3 [41].

Carbon nanotubes (CNTs) are ultrathin cylinders of an *sp*^2^-bonded hexagonal network of carbon atoms [1] (Figure 1(a)) and have attracted much attention in various fields due to their excellent intrinsic optical, mechanical, electronic, and thermal properties [2], [3], [4], [5], [6], [7], [8], [9], [10]. Previous experiments using individual single- or multi-walled CNTs have demonstrated high electrical [2, 3] and thermal conductivity [4, 5] owing to the suppressed scattering of electrons and phonons, an exceptional strength-to-weight ratio [6, 7], and high thermal stability [8, 9]. In addition, the strong light–matter interaction of CNTs [10, 11], which is robust even at more than 2000 K [9], is promising not only for photonic but also for thermo-optic applications. Many of the physical properties of CNTs, including their semiconducting or metallic nature [12], [13], [14], optical resonance energy [15], [16], [17], and ultimate tensile strength [7, 18], strongly depend on their chiral structure (chirality), specified by a pair of diameters and wrapping (chiral) angles or chiral indices (*n*, *m*) (Figure 1(b)). Therefore, the preferred structure of nanotubes depends on the applications, and the fabrication of single-chirality nanotube assemblies that clearly indicates the structure-specific physical properties is desired.

Although the large-scale synthesis of nanotubes with only single chirality remains difficult, recent progress on methods for separating nanotubes with a specific chirality from a mixed sample containing nanotubes with various chiral structures [19], [20], [21], [22], [23], [24], [25], [26], [27], [28], [29] has made it possible to use single-chirality nanotubes to fabricate macroscopic nanotube assemblies. In an assembly of mixed nanotubes, it is generally difficult to retain many of the excellent properties of individual nanotubes due to structural inhomogeneity [30] and/or nanotube–nanotube interactions [31]. In contrast, the distinct optical properties of individual nanotubes can be fairly well preserved in a nearly single-chirality nanotube assembly [32], [33], [34], [35], [36], [37]. This is because exciton states [38], [39], [40], which are hydrogen-like bound states of electrons and holes, and are thermally robust in CNTs due to the large binding energy [38], [39], [40] exhibit strong light–matter interactions within a narrow spectral window (Figure 1(c)).

Because the exciton resonance energies depend on the chirality [15], [16], [17], only assemblies of single-chirality CNTs can exhibit distinct color [42] due to sharp optical resonance (Figure 1(d)). The color of CNTs is often thought to be black based on the association with the color of conventional carbon graphite materials; however, single-chirality nanotube assemblies exhibit a variety of colors, including blue, green, and red (Figure 1(e)). They are particularly attractive as optofunctional materials, and have provided opportunities to observe novel physical phenomena, including exciton–polariton effects [33], [34], [35] and high-harmonic generation [37]. For researchers and engineers who wish to design photonic devices using single-chirality nanotubes, knowledge of the complex refractive index spectra of the nanotubes, including their chirality and carbon density dependence is necessary, as this information enables the prediction of reflection, transmission, and absorption in the medium [43, 44]. The complex refractive indices of individual SWCNTs as a function of photon energy have been reported [45]. However, the complex refractive index spectra of various single-chirality nanotube assemblies are scarce in the literature; only those of a specific chirality in a limited band (visible to near-infrared) are currently available [33]. Therefore, comprehensive knowledge of the broadband complex refractive index spectra and their chirality dependence is strongly desired to facilitate the design of macroscale photonic devices that can operate in various desired wavelength regions using single-chirality nanotube assemblies.

In this paper, we report the broadband complex refractive index spectra of single-chirality CNT assemblies in the mid-infrared to visible wavelength region. Single-walled carbon nanotubes (SWCNTs) were separated using the gel chromatography technique [28, 29]. We fabricated SWCNT membranes via filtration of SWCNT solutions and transferred them onto sapphire substrates (hereafter referred to as *on-sapphire*) or over metal washers to keep the membranes suspended in the air (hereafter referred to as *free-standing*). To determine the complex refractive index spectra of the SWCNT membranes, the thicknesses of the membranes were determined using a stylus profilometer, and reflection and transmission spectra were measured in the photon energy range of 0.06–3.10 eV using a Fourier transform infrared spectrometer (for the mid-infrared to near-infrared range) and a homemade optical setup (for the visible range; Supplementary figure 1). The complex refractive index spectra were obtained by reproducing all the experimental optical spectra using model functions considering the optical susceptibilities of longitudinal excitons, excitonic phonon sidebands [46, 47], the background response (phenomenologically including the effects of the nonresonant energy continuum [48] and transverse excitons [49, 50]), and the Drude-like response. At the first subband (*S*_11_) exciton resonance, the real and imaginary parts of the complex refractive indices of an SWCNT membrane with a diameter of 0.92 nm and bulk density of 1 g cm^−3^ were determined as 3.3 and 2.2i, respectively, while they were 2.0 and 1.1i at the second subband (*S*_22_) exciton resonance, respectively. From these observations, we further obtained a ready-to-use empirical formula to reproduce the complex refractive index spectra of SWCNTs with various chiral structures. This can pave the way for the straightforward design of optical/photonic devices using various types of single-chirality nanotube membranes.

## 2 Results and discussion

**Figure 2:**
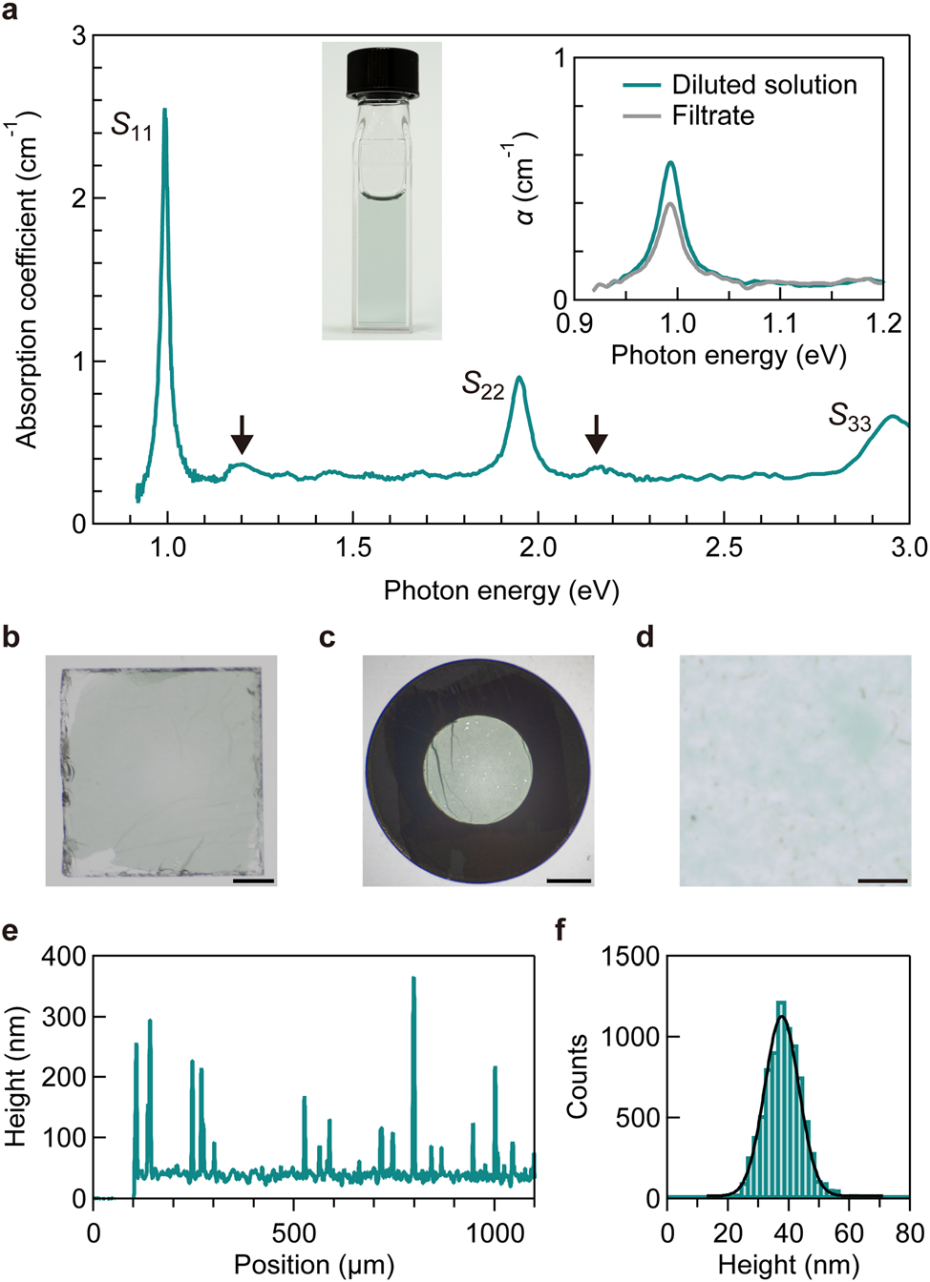
Characterization of CNT membrane. (a) Absorption spectrum of an SWCNT solution with (10,3) chirality. *S*_*jj*_ indicates the *j*th subband exciton resonance. The black arrows indicate the phonon sidebands originating from the optical longitudinal phonons. The left inset contains a photograph of the sample, while the right inset presents the absorption coefficient (*α*) spectra of the diluted solution (green) and filtrate (gray). (b and c) Photographs of an on-sapphire (b) and free-standing (c) SWCNT membrane with (10,3) chirality. Scale bar, 1 mm. (d) Optical microscope image of the on-sapphire SWCNT membrane. Scale bar, 10 µm. (e and f) Height profile (e) and height histogram (f) of the SWCNT membrane on a silicon substrate. The black solid curve is the fitting result using the Gaussian function with an average thickness of 37 nm and a standard deviation of 6 nm.

Single-chirality SWCNT solutions were prepared using the gel chromatography separation method [28, 29] (see Methods in Supplementary information). Figure 2(a) displays the absorption spectrum of a solution sample containing (10,3) SWCNTs. Several peaks and continuous absorption features can be observed. The highest and sharpest peak observed at 0.99 eV is assigned to the *S*_11_ exciton, while the second and third highest peaks at 1.95 and 2.96 eV are the exciton resonances of the *S*_22_ and third (*S*_33_) subband, respectively. The light green color of the SWCNT solution (photograph in Figure 2(a)) is ascribed to the strong *S*_22_ excitonic absorption of red light. There are weak absorption peaks in addition to this strong excitonic absorption. The peaks at approximately 0.2 eV above the *S*_11_ and *S*_22_ absorption (indicated by black arrows in Figure 2(a)) are the excitonic phonon sidebands originating from the scattering of excitons with longitudinal optical phonons near the Γ and K points of the graphene Brillouin zone [46, 47]. The featureless continuum absorption band above the *S*_11_ exciton energy is attributed to the contributions of various transitions, including the exciton continuum [48], cross-polarized interband absorption accompanying a change in the angular momentum along the SWCNT axis [49, 50], and a small amount of residual SWCNTs with other chiralities.

On-sapphire and free-standing SWCNT membranes were fabricated via filtration of single-chirality SWCNT solutions. Figure 2(b) and (c) presents images of an on-sapphire and a free-standing SWCNT membrane, respectively. The green color of the membranes indicates that the major optical absorption of the individual (10,3) SWCNTs observed in the solution was retained in the membrane. Figure 2(d) presents an optical microscope image of the on-sapphire SWCNT membrane. The surface appears clean, and no large impurities are observed. Figure 2(e) presents the typical height profile of the SWCNT membrane placed on a silicon substrate. The height profile of the SWCNT membrane was measured with respect to the surface of the silicon substrate located at a position below ∼100 µm. Although several spikes presumably due to residual impurities can be observed, the membrane exhibits almost the constant thickness in the wide range (>1 mm), and the thickness is much smaller than the visible light wavelength. Figure 2(f) presents the height histogram of the SWCNT membrane together with the fitting results using a Gaussian function (solid black curve). The fitting analysis reveals an average thickness of 37 nm with a standard deviation of 6 nm. Thus, the SWCNT membrane can be regarded as an optically flat surface in the wavelength range covered in this study.

To characterize the SWCNT membrane more quantitatively, we estimated the bulk density of the (10,3) SWCNT membrane as follows. Initially, the volume of the SWCNT membrane was estimated. The SWCNT membrane was regarded as a thin disk whose bottom area and height were estimated as 2.1 cm^2^ (effective filtration area of the filter holder) and 37 nm (thickness), respectively. Thus, the volume of the membrane was estimated as 7.8 × 10^−6^ cm^3^. Then, the mass of the SWCNT membrane was estimated. The inset of Figure 2(a) presents the absorption spectra of the SWCNT solution (green) and the filtrate (gray), in which the SWCNT solution was diluted to avoid surfactant precipitation. In the process of membrane fabrication, some SWCNTs in the solution were captured by the filter while other SWCNTs remained in the filtrate. Assuming that the difference in the net mass of the SWCNT between the raw SWCNT solution and the filtrate was equal to the mass of the SWCNT membrane remaining on the filter, the mass of the membrane was estimated as 7 µg. Consequently, the bulk density of the (10,3) SWCNTs in the membrane was deduced as 1 g cm^−3^ from the volume and mass. The densities of the other membranes estimated using the same method were also 1 g cm^−3^ (see Supplementary note 1 for discussion on volume filling factor).

**Figure 3:**
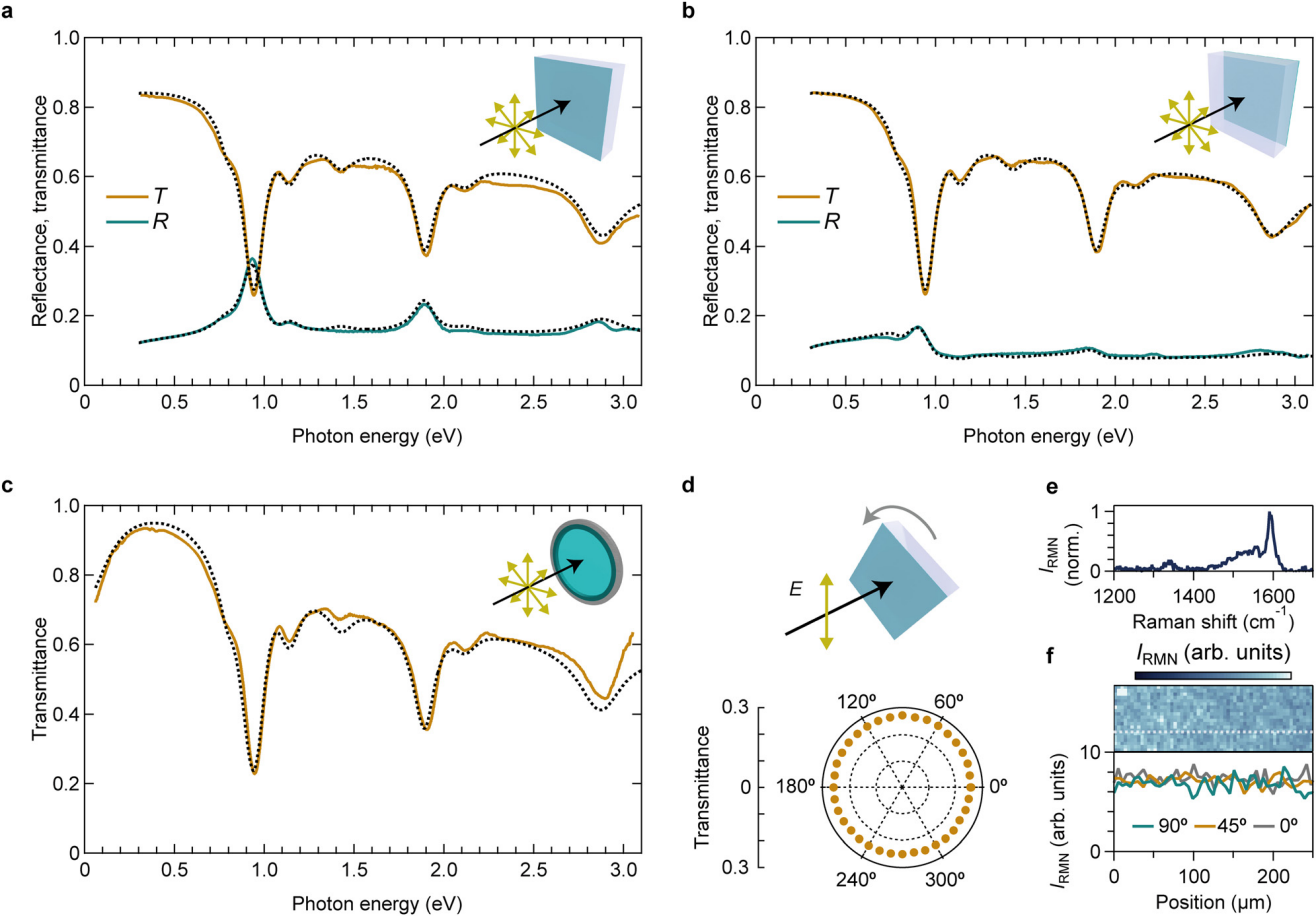
Optical spectra of CNT membranes. (a and b) Transmittance (*T*) and reflectance (*R*) spectra of the on-sapphire membrane composed of (10,3) SWCNTs; the probe light incident on the SWCNT (a) and sapphire (b) sides. (c) Transmittance spectra of the free-standing membrane. The insets of (a)–(c) present schematics of the configurations of the samples and light. The dotted curves represent the calculation results. (d) Schematic of a setup for polarization-dependent transmittance (upper panel) and a polar plot of the transmittance of the on-sapphire membrane at the first subband exciton peak (bottom panel). (e) *G*-mode feature of the Raman spectrum. (f) *G*-mode intensity map of the on-sapphire membrane (upper panel) and the polarization dependence of the *G*-mode intensity along the white dotted line in the upper panel (bottom panel). *I*_RMN_ denotes the Raman intensity.

Figure 3(a)–(c) presents the optical spectra of the on-sapphire SWCNT and the free-standing membrane (see Methods in Supplementary information). The insets display schematics of the experimental configurations. The optical spectra of the on-sapphire membrane were measured in two configurations, where the incident light entered from either the side of the SWCNT membrane or the sapphire substrate (insets of Figure 3(a) and (b)). The measurement region was limited within a spot with a diameter of 3 mm using a holder (Supplementary figure 1); thus the spectra reflect the averaged responses over the macroscopic region with the area of ∼7 mm^2^. All the transmittance (orange curves) spectra exhibit excitonic peaks and their phonon sidebands. These spectra mainly reflect the absorption properties. The peaks exhibit redshifts and broadening compared to those in the absorption spectra of the solution. A minor shoulder peak at approximately 0.8 eV is assigned to small amount of residual SWCNTs with other chiralities contained in the single-chirality SWCNT sample. The SWCNT side of the on-sapphire membrane exhibits a relatively large reflectance of ∼0.4 at the *S*_11_ exciton resonance energy (green curve in Figure 3(a)). The reflectance spectra of the on-sapphire membrane irradiated from the SWCNT side (Figure 3(a)) exhibit more distinctive exciton peaks than that from the sapphire side (Figure 3(b)), which indicates that the refractive index difference at the air–SWCNT membrane interface is larger than that at the sapphire–SWCNT membrane interface. The transmittance spectrum of the free-standing membrane (Figure 3(c)) allows obtaining information in the photon energy below ∼0.3 eV for which the strong absorption by the substrate prohibits acquisition of the transmittance spectra of the on-sapphire membrane. As the photon energy decreases below ∼0.3 eV, the transmittance of the free-standing membrane is reduced. We inferred that the origin of this low-energy feature is the Drude response of unintentionally doped free carriers.

We also studied the in-plane orientation of SWCNTs in the membrane using polarization spectroscopy. The polar plot in Figure 3(d) illustrates the polarization dependence of the transmittance at the *S*_11_ exciton peak (its configuration is indicated in the schematic); the transmittance is almost independent of the polarization angle. Since each individual SWCNT strongly exhibits anisotropic optical responses, the results suggest the random in-plane orientation of SWCNTs. To examine the local orientation of SWCNTs more microscopically than by transmission spectroscopy, we used polarized Raman imaging spectroscopy. Figure 3(e) presents a typical Raman spectrum of the SWCNT membrane, and the peak around 1590 cm^−1^ is the *G*-mode feature. The upper panel of Figure 3(f) presents the *G*-mode intensity map of the on-sapphire membrane, while the bottom panel of Figure 3(f) presents the *G*-mode intensities along the white dotted line under various excitation polarization conditions. There is no clear polarization dependence. These results demonstrate that the in-plane orientation of SWCNTs in the membrane is random.

**Figure 4:**
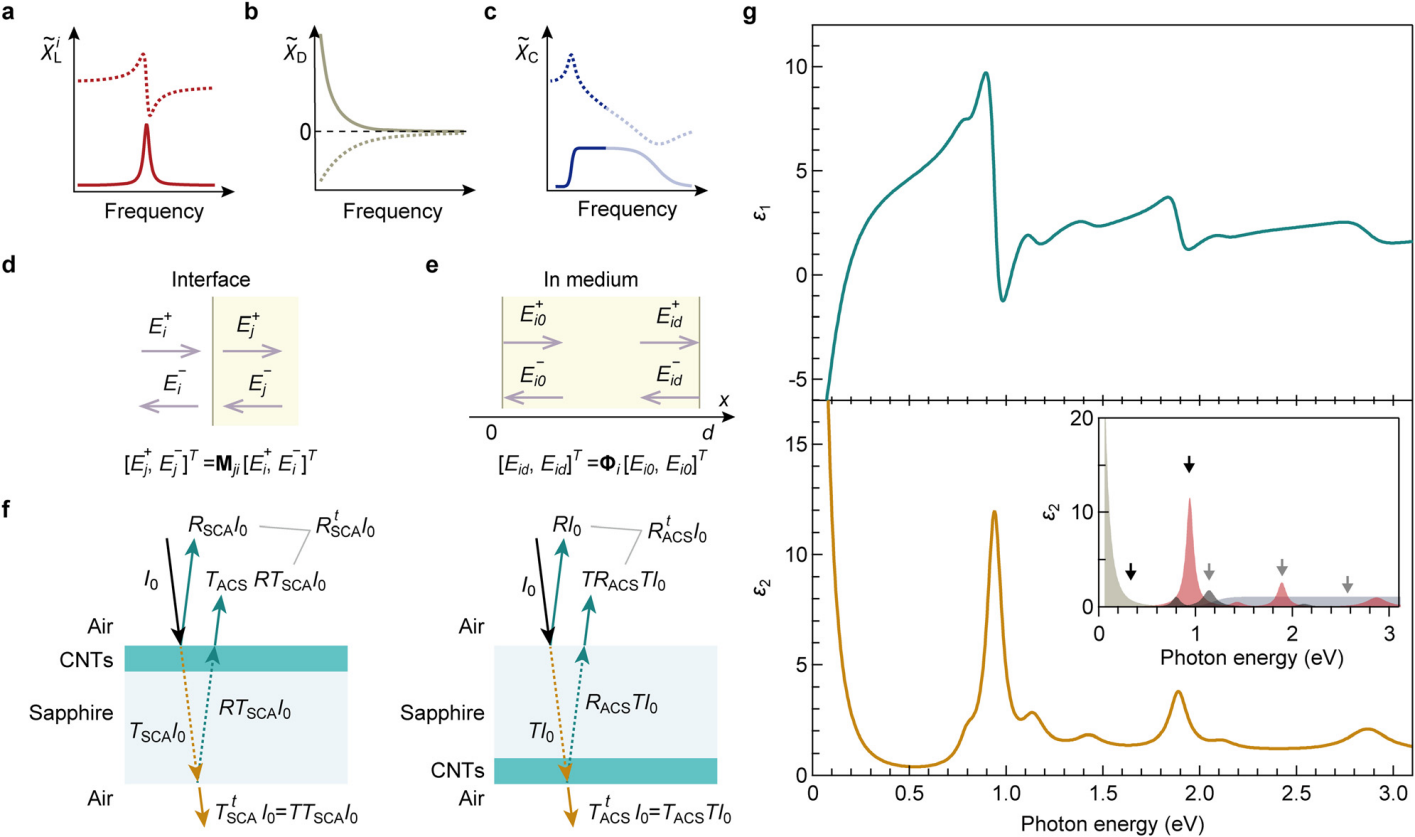
Model optical susceptibilities. (a)–(c) Schematics of the complex susceptibility of the Lorentz model ${\tilde{\chi }}_{\text{L}}^{i}$ (a), Drude model ${\tilde{\chi }}_{\text{D}}$ (b), and phenomenological model for the continuum energy band ${\tilde{\chi }}_{\text{C}}$ (c). Solid and dotted curves represent the imaginary and real parts, respectively. (d and e) Transfer matrix $M_{ji}$ (d) and propagation matrix $\Phi_{i}$ (e) that relate two-component vectors ${\left[E_{i\left(j,i0,id\right)}^{+},E_{i\left(j,i0,id\right)}^{-}\right]}^{T}$ at different positions. (f) Reflection (green solid arrows) and transmission (orange solid arrows) of an on-sapphire CNT membrane. Probe light with intensity *I*_0_ (black solid arrows) is incident on the CNT side (left panel) and sapphire side (right panel). Optical paths are drawn in oblique incidence configurations for clarity. *R*(*T*)_*kji*_ is the reflectance (transmittance) when the probe light propagates in the order of media *i*, *j*, and *k*, where subscripts C, S, and A denote the CNT membrane, sapphire, and air, respectively. R(T) is the reflectivity (transmissivity) at the interface between the sapphire substrate and air. (g) Complex dielectric function $\left(\tilde{\epsilon }=\epsilon_{1}+i\epsilon_{2}\right)$ of the membrane composed of (10,3) CNTs. The inset presents the imaginary part of the dielectric function of excitons and their phonon sidebands (red), Drude-like response (gray), and continuous energy band (blue).

Here, we determine the complex refractive index spectra, which are consistent with all optical spectra presented in Figure 3. Initially, we model the macroscopic optical susceptibility functions of the membranes per bulk density of carbon atoms. Because excitonic resonance spectra of individual SWCNTs are well reproduced using the Lorenz model [7, 10, 51], we approximately express all optical responses arising from excitons and their phonon sidebands in the SWCNT membrane per unit bulk density using the simple Lorenz model of the form ${\tilde{\chi }}_{\text{L}}^{i}\left(\omega \right)=f_{\text{L}}^{i}{\left[\left(\omega_{\text{L}}^{i2}-\omega^{2}\right)-i\omega \gamma_{\text{L}}^{i}\right]}^{-1}$, where $f_{\text{L}}^{i}$, $\omega_{\text{L}}^{i}$, and $\gamma_{\text{L}}^{i}$ are the strength, resonant frequency, and damping term for *i*th Lorentz oscillator, respectively (Figure 4(a)). Although the exciton resonance peaks of the membranes are broader than their counterparts for the individual SWCNTs, the Lorentz model can still sufficiently reproduce these peaks, as illustrated in Figure 3. This implies that the peak broadening is mainly caused by the reduced exciton dephasing time due to bundling rather than to inhomogeneity. The decrease in transmission below ∼0.3 eV is described assuming the Drude-like response (Figure 4(b)) per unit bulk density with the conventional form ${\tilde{\chi }}_{\text{D}}\left(\omega \right)=-A_{\text{D}}{\left[\omega \left(\omega +i\gamma_{\text{D}}\right)\right]}^{-1}$, where $A_{\text{D}}$ and $\gamma_{\text{D}}$ are the strength and damping term, respectively. Additionally, we introduce the phenomenological optical susceptibility function ${\tilde{\chi }}_{\text{C}}\left(\omega \right)$ to mimic the nearly featureless continuum band in the photon energy region above the *S*_11_ exciton resonance with referring to the previous works [48], [49], [50, 52] (Figure 4(c), see Supplementary note 2 for the details). Using these functions, the complex optical susceptibility ($\tilde{\chi }$) for an SWCNT membrane with a bulk density of ρ are modeled as

(1) $$\tilde{\chi }\left(\rho ,\omega \right)=\rho \left[{\tilde{\chi }}_{\text{C}}\left(\omega \right)+{\tilde{\chi }}_{\text{D}}\left(\omega \right)+\chi_{\text{B}}+\underset{i}{\sum }{\tilde{\chi }}_{\text{L}}^{i}\left(\omega \right)\right]$$

where $\chi_{\text{B}}$ is the background susceptibility (real constant) describing all contributions other than those taken into account by ${\tilde{\chi }}_{\text{C}}$, ${\tilde{\chi }}_{\text{D}}$, and ${\tilde{\chi }}_{\text{L}}^{i}$. The complex relative dielectric function $\left(\tilde{\epsilon }=\epsilon_{1}+i\epsilon_{2}\right)$ and the complex reflective index ($\tilde{n}=n+i\kappa$) are given through $\tilde{n}\left(\rho ,\omega \right)=\sqrt{\tilde{\epsilon }\left(\rho ,\omega \right)}=\sqrt{1+\tilde{\chi }\left(\rho ,\omega \right)}$.

The reflectance and transmission spectra are calculated using the optical transfer matrix technique [44] (see Supplementary note 3 for the details). This scheme describes light propagation in a medium *i* using a two-component vector $E_{i}={\left(E_{i}^{+},E_{i}^{-}\right)}^{T}$, where $E_{i}^{+\left(-\right)}$ is the amplitude of an electric field of forward (backward) propagating light (Figure 4(d) and (e)). When light enters from medium *i* to medium *j*, the transfer matrix $\left(M_{ji}\right)$ for normal incidence describes the relationships between the two-component vectors adjacent to an interface between media *i* and *j* through $E_{j}=M_{ji}E_{i}$ (Figure 4(d)). The propagation matrix $\left(\Phi_{i}\right)$ describes the amplitude and phase change in the two-component vector in the propagation in medium *i* with a thickness of *d* (Figure 4(e)). When light propagates in the order of media *i*, *j*, and *k*, the entire optical transfer matrix is given by $G_{kji}=M_{kj}\Phi_{j}M_{ji}$. The reflectance and transmittance are given by $R_{kji}={\left|G_{kji}^{21}/G_{kji}^{22}\right|}^{2}$ and $T_{kji}=n_{k}/n_{i}{\left|G_{kji}^{11}-G_{kji}^{12}G_{kji}^{21}/G_{kji}^{22}\right|}^{2}$, where the superscripts represent the matrix elements of $G_{kji}$. In the analysis of the optical spectra of the on-sapphire SWCNT membrane, we account for reflectivity (R) and transmissivity (T) at the single interface between air and the sapphire substrate, and it should be noted that these values are the same regardless of the direction in which the light travels. The total reflectance ($R_{\text{SCA}}^{t}$, $R_{\text{ACS}}^{t}$) and transmittance ($T_{\text{SCA}}^{t}$, $T_{\text{ACS}}^{t}$) of the on-sapphire membrane are expressed by

$$R_{\text{SCA}}^{t}=R_{\text{SCA}}+T_{\text{ACS}}RT_{\text{SCA}},\;T_{\text{SCA}}^{t}=TT_{\text{SCA}}$$

(2) $$R_{ACS}^{t}=R+TR_{ACS}T,\;T_{\text{ACS}}^{t}=T_{\text{ACS}}T$$

where subscripts S, C, and A represent the sapphire substrate, CNT membrane, and air, respectively (Figure 4(f)). $R_{\text{SCA}\left(\text{ACS}\right)}^{t}$ and $T_{\text{SCA}\left(\text{ACS}\right)}^{t}$ are the total reflectance and transmittance in the configuration of light incident on the SWCNT (sapphire) side. The transmittance of the free-standing membrane is simply given by $T_{\text{ACA}}$.

The black dotted curves in Figure 3(a)–(c) represent the calculation results to reproduce all the optical spectra in the photon energy range of 0.06–3.10 eV obtained by a global fitting procedure. The calculated spectra well reproduce all the experimental spectra. Figure 4(g) presents the obtained complex dielectric function of the SWCNT membrane, $\tilde{\epsilon }=\epsilon_{1}+i\epsilon_{2}$, which is consistent with all the experimentally obtained optical spectra. The obtained fit parameters are summarized in Supplementary table 1. The inset displays the imaginary parts of the dielectric functions of each component. The *S*_11_ exciton and Drude-like response (black arrows) largely contribute to the entire dielectric constant of the SWCNT membrane, followed by the *S*_11_ exciton phonon sideband, the *S*_22_ exciton, and the continuous absorption band (gray arrows).

**Figure 5:**
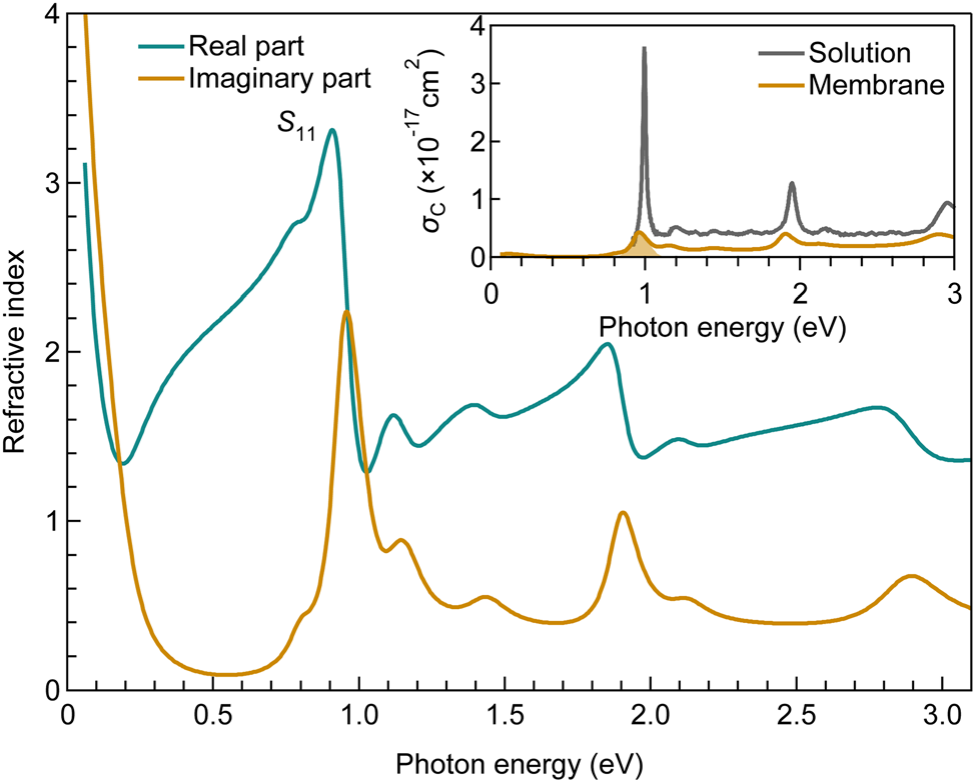
Complex refractive index spectrum of CNT membrane with (10,3) chirality. The inset displays the absorption cross section per carbon atom (*σ*_C_) of the membrane (orange) and the raw solution (gray).

Finally, as illustrated in Figure 5, we obtained the model complex refractive index spectrum of the (10,3) SWCNT membrane that closely reproduces all the measured optical spectra using $\tilde{n}\left(\rho ,\omega \right)=\sqrt{\tilde{\epsilon }\left(\rho ,\omega \right)}$. In the photon energy above 0.2 eV, both the real and imaginary parts have maximum values around the *S*_11_ exciton resonance (3.3 and 2.2i, respectively), while they are 2.0 and 1.1i at the *S*_22_ exciton resonance, respectively. The real part at the *S*_11_ exciton resonance reaches approximately double the average refractive index (∼1.5) above the *S*_11_ exciton resonance. The inset presents the absorption cross section of carbon atoms (*σ*_C_) for the membrane and the raw solutions estimated from the absorption coefficient divided by the number of carbon atoms. The SWCNT membrane exhibits a value of 4.3 × 10^−18^ cm^2^ at the *S*_11_ exciton resonance peak, which is approximately 1/8 of that of the solution. This is partially attributed to the linewidth broadening because the integrated absorption cross section for the *S*_11_ exciton resonance in the membrane is kept as high as approximately 50% of that in the solution sample. This result indicates that SWCNTs retain the substantial intrinsic oscillator strength of the *S*_11_ exciton in the membrane (see Supplementary note 4 for further discussion).

**Figure 6:**
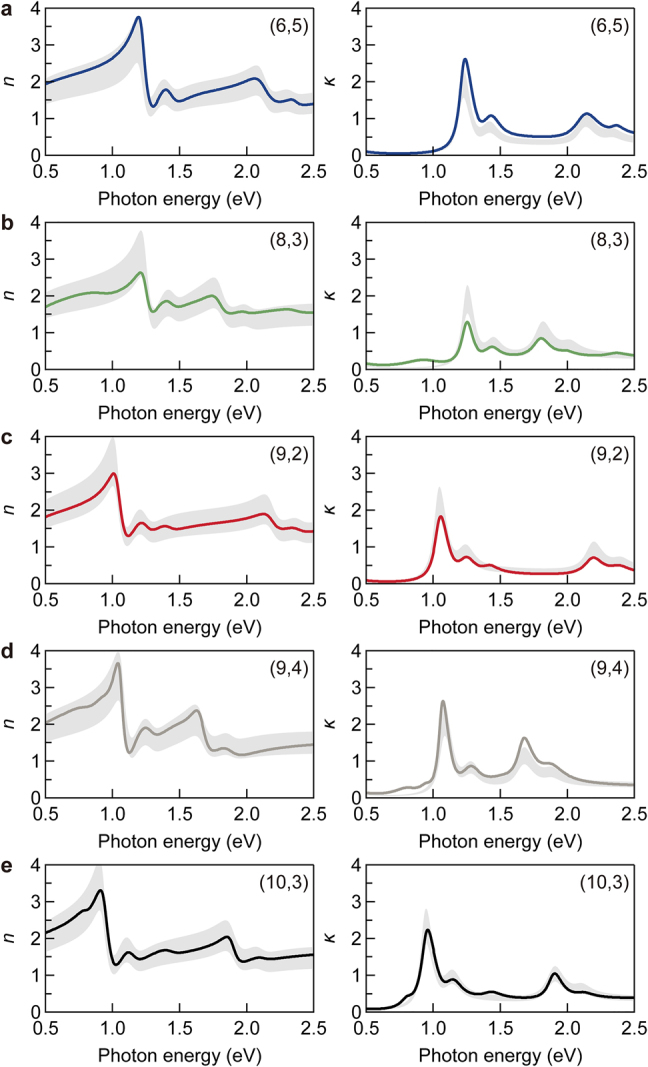
Complex refractive index spectra of single-chirality SWCNT membrane. (a)–(e) Chiralities are (6,5) (a), (8,3) (b), (9,2) (c), (9,4) (d), and (10,3) (e). Left and right panels display the real (refractive index, n) and imaginary (extinction coefficient, κ) parts, respectively. The bulk densities of the membranes are 1 g cm^−3^. The shaded areas indicate the calculation results derived from the empirical formula.

Using a similar procedure to that for the (10,3) SWCNT membrane, we obtained the complex refractive index spectra from the reflection and transmission measurements of the on-sapphire membranes consisting of SWCNTs with other chiral structures. Figure 6 summarizes the complex refractive index spectra of (6,5), (8,3), (9,2), (9,4), and (10,3) SWCNT membranes with ρ=1 g cm^−3^ (see Supplementary figures 2–5 and tables 2–5). The real and imaginary parts of the refractive indices at the *S*_11_ exciton resonance were determined to be approximately 2.7–3.6 and 1.3i–2.4i, respectively. Although the peak positions are different owing to the chirality-dependent exciton resonance energy, the complex refractive index spectra exhibit similar behavior. This inspired us to develop a ready-to-use empirical formula to represent the complex refractive index spectrum of an SWCNT membrane with arbitral chirality for engineering purposes.

In the photon energy range of 0.5–2.5 eV, the main contributions are the following six components: *S*_11_ and *S*_22_ exciton states and their phonon sidebands located at ∼0.2 eV above the excitons, the continuous energy band, and the Drude-like response. We took averages of all the measured chiral structures to obtain representative values for the following quantities: the strength $\left(f_{\text{L}}^{i}\right)$ and damping term $\left(\gamma_{\text{L}}^{i}\right)$ in each Lorentz oscillator $\left({\tilde{\chi }}_{\text{L}}^{i}\right)$, the strength $\left(A_{\text{D}}\right)$ and damping $\left(\gamma_{\text{D}}\right)$ in the Drude-like response $\left({\tilde{\chi }}_{\text{D}}\right)$, the absorption strength $\left(A_{\text{C}}\right)$ and gradient $\left(\gamma_{\text{C}}^{-1}\right)$ in the continuum energy band (${\tilde{\chi }}_{\text{C}}$; see Supplementary note 2 for the details), and the deviations of the resonant frequencies ($\omega_{\text{L}}$, $\omega_{\text{C}}$) in the membrane from those of individual air-suspended SWCNTs previously reported in the literature [17] (see Supplementary table 6). The gray shaded area in Figure 6 represents the results calculated using these obtained average values with a ±20% error band, and most of the experimental spectra are contained within the gray shaded area. Based on these considerations, we provide an empirical formula of the complex refractive index of a single-chirality SWCNT membrane with a random in-plane nanotube orientation as

(3) $$\tilde{n}\left(\rho ,\omega \right)=\sqrt{1+\rho \left[{\tilde{\chi }}_{\text{C}}\left(\omega \right)+{\tilde{\chi }}_{\text{D}}\left(\omega \right)+〈\chi_{\text{B}}〉+\sum_{i=1}^{4}{\tilde{\chi }}_{\text{L}}^{i}\left(\omega \right)\right]}$$

with the parameters listed in Supplementary table 6 ($\chi_{\text{B}}$ is the averaged value of the background susceptibility). This empirical formula is particularly useful in designing SWCNT-based optical devices with a desired cut-off photon energy in selective photon absorber and thermal light emitter [9, 11]. The complex refractive indices of various SWCNTs can also be used in designing devices using both SWCNTs and conventional dielectric materials, such as dielectric multilayer filters.

Here, we briefly comment on the applicability and limitations of this empirical formula. The formula can be applied to membranes composed of SWCNTs with random in-plane orientation, and correction is required for membranes with aligned SWCNTs [32] or composite materials. The formula was derived from experiments using five types of semiconducting SWCNTs whose diameters are in the range of 0.75–0.92 nm. Thus, this empirical formula is applicable to membranes of SWCNTs with similar diameters. These SWCNTs have only *S*_11_ and *S*_22_ exciton states in the photon energy range of 0.5–2.5 eV [17]. At the present stage, small deviations of the imaginary parts are observed around the *S*_11_ exciton state for the (6,5) and (8,3) SWCNTs (Figure 6). This may be attributed to the fact that we neglected the chirality dependence of the exciton oscillator strength [53]. Since it was difficult to address this issue experimentally under the precision of the bulk density in this study, this topic is left for future research.

## 3 Conclusions

In summary, we studied the complex refractive index spectra of SWCNT membranes composed of single-chirality species in the photon energy range of 0.06–3.10 eV. We measured the reflectance and transmittance spectra of the SWCNT membranes, and successfully reproduced them using model functions that take major contributions to the optical susceptibility into account, including the optical susceptibilities of longitudinal excitons, excitonic phonon sidebands, the phenomenological continuum energy band, the Drude-like response, and the nonresonant background response. The complex refractive index spectra of the five types of SWCNT membranes were determined. From these complex refractive index spectra, we obtained an empirical formula that reproduces the complex refractive index spectra of membranes composed of semiconducting single-chirality SWCNTs. The applicability of the formula was verified for SWCNTs within the diameter range of 0.75–0.92 nm. This information will be useful for designing various photonic devices using single-chirality SWCNT assemblies.

## Supplementary Material

Supplementary Material
