# Supplementary material for: Empirical formulation of broadband complex refractive index spectra of single-chirality carbon nanotube assembly
Source: Nanophotonics. 2022 Jan 12;11(5):1011–20. doi: 10.1515/nanoph-2021-0728 (PMC11501240; doi:10.1515/nanoph-2021-0728)
Supplement: Supplementary file 1 — Supplementary Material [file j_nanoph-2021-0728_suppl.pdf]

Supplementary information

Empirical formulation of broadband complex refractive index spectra of single-chirality carbon nanotube assembly

Taishi Nishihara,[1] Akira Takakura,[1] Masafumi Shimasaki,[1] Kazunari Matsuda,[1] Takeshi Tanaka,[2] Hiromichi Kataura,[2] and Yuhei Miyauchi[1]

[1]*Institute of Advanced Energy, Kyoto University, Uji, Kyoto 611-0011, Japan.*

[2]*Nanomaterials Research Institute, National Institute of Advanced Industrial Science and Technology (AIST), Tsukuba, Ibaraki 305-8565, Japan.*

Methods

Fabrication of SWCNT membranes.

We fabricated single-chirality SWCNT membranes using a vacuum filtration method, referring to Ref. 1. Single-chirality SWCNTs were separated from the starting materials of CoMoCAT (SG65, Sigma-Aldrich) and HiPCo (NanoIntegris) nanotubes by the gel column chromatography method [2,3]. The separated SWCNTs were dispersed in pure water with various surfactants, including sodium deoxycholate (DOC), sodium cholate (SC), sodium dodecyl sulfate (SDS), and sodium lithocholate (LC). We prepared five solutions composed of the (6,5) SWCNTs (SG65, 1.6 μg mL^{-1} in 0.5% SC + 0.5% SDS + 0.03% DOC) [2], the (8,3) SWCNTs (HiPCo, 0.12 μg mL^{-1} in 0.3% SC + 0.9% SDS + 0.1% LC) [3], the (9,2) SWCNTs (HiPCo, 0.1 μg mL^{-1} in 0.3% SC + 0.9% SDS + 0.12% LC) [3], the (9,4) SWCNTs (HiPCo, 0.87 μg mL^{-1} in 0.5% SC + 1% SDS + 0.155% DOC) [2], and the (10,3) SWCNTs (HiPCo, 1.4 μg mL^{-1} in 0.5% SC + 1% SDS + 0.155% DOC) [2]. For vacuum filtration, we used a polycarbonate membrane filter with a pore size of 100 nm (MERCK, VCTP02500) and the filter holder with an effective filtration area of 2.1 cm^2 (ADVANTECH, KGS-25). The diluted dispersed SWCNT solution with a surfactant concentration below the critical micelle concentration of each surfactant (0.08–0.25% wt./vol. for DOC, 0.39–0.65% wt./vol. for SC, and 0.20–0.29% wt./vol. for SDS) was filtered at a pressure of 50–80 kPa for approximately 30 min. Following the SWCNT solution, hot water (5 mL) was poured into the filtering system to remove excess surfactants. Then, the membrane filter with the SWCNTs was dried by air at a pressure of 1–3 kPa for 30 min. The obtained SWCNT membrane on a polycarbonate membrane filter was cut to a size suitable for transfer onto substrates (sapphire for spectroscopy and silicon for thickness measurements) or stainless washers supporting free-standing membranes. The SWCNT membrane with a polycarbonate membrane filter was immersed in chloroform for 15 min to dissolve the membrane filter. The SWCNT film floating on chloroform was scooped using the substrate or washer and was cleaned using chloroform, followed by ethanol and acetone.

Thickness measurement and reflection, transmission, and Raman spectroscopy.

The thickness of the SWCNT membranes was measured using a stylus profilometer (Dektak150, BRUKER). Broadband optical spectroscopy in the photon energy range of 0.06–3.10 eV was performed using the combination of a FTIR system (FT/IR-6600, JASCO) and a homemade optical system (Supplementary Figure 1). The FTIR system covered the far-infrared to near-infrared wavelength region (0.06–1.77 eV). The homemade optical system consisted of a halogen lamp and a fiber-coupled spectrometer (Ocean Optics USB2000+) or a monochromator attached to a thermoelectrically cooled charge-coupled device camera (Princeton Instruments, ProEM). The light sources used in these optical measurements were unpolarized, and the incident angle was set to 10° for the reflection measurements. A simple calculation using Fresnel equations demonstrates that reflectivity for unpolarized light, which is given by the average of the reflectivity for *s*- and *p*-polarized light, is almost independent of the incident angles in the range of 0–10°. Thus, for simplicity, we treated the incident angle as 0° for spectral analysis of reflectance. The microscopic morphology of the SWCNT membranes was studied using a micro-Raman setup (Nanophoton, RAMANtouch), where the sample membranes were placed in a nitrogen gas atmosphere to avoid photo-induced degradation.

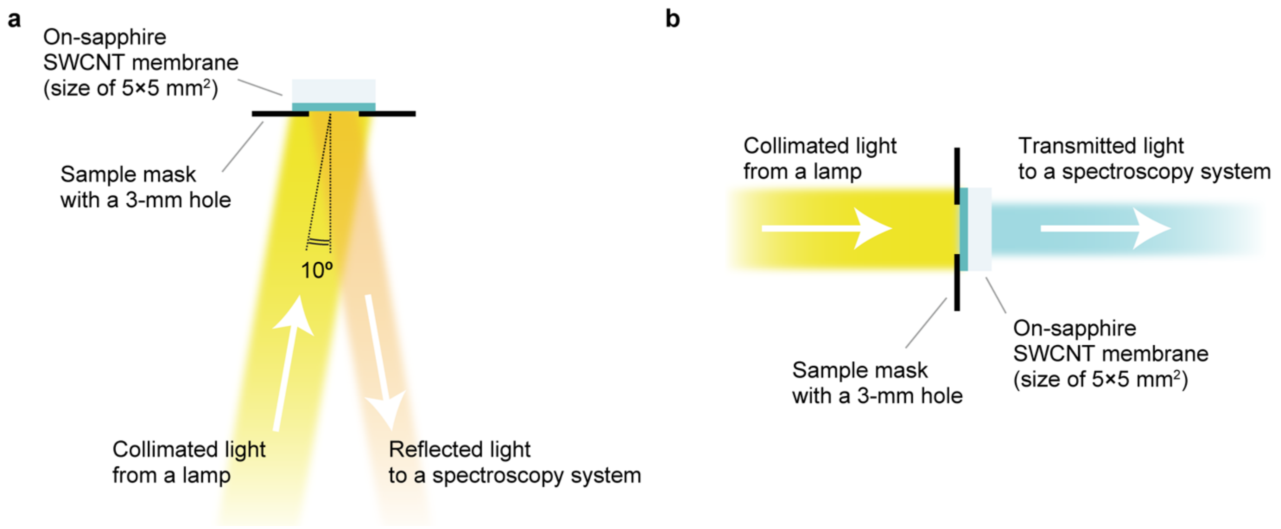

Supplementary Figure 1: Schematics of the optical setup around samples. Reflection (a) and transmission (b) spectroscopies.

Supplementary Note 1: Volume filling factor of carbon nanotubes in membranes

Volume filling factor of single-walled carbon nanotubes (SWCNTs) in a membrane was estimated as follows. Assuming that an SWCNT is a filled cylinder with shell thickness of inter-layer graphite separation (0.34 nm) [4], volume filling factor of (10,3) SWCNTs in a membrane with a bulk density of 1 g cm^{-3} is estimated as ~0.57. This value is ~60% of that of closest packed structure of cylinders (0.91), and the SWCNTs occupied about half the volume of the membrane.

Supplementary Note 2: Phenomenological optical susceptibility function to mimic the nearly featureless continuum band

We introduce the phenomenological optical susceptibility function $\tilde{\chi}_{\mathrm{C}}(\omega)$ to mimic the nearly featureless continuum band in the photon energy region above the S_{11} exciton resonance (Figure 4c). The imaginary part of $\tilde{\chi}_{\mathrm{C}}(\omega)$, which is related to absorption mainly owing to the exciton continuum [5] and transverse exciton absorption [6,7], is roughly approximated using a single broadened step-function-like form for analytical convenience. This feature is well described using a series of $\pi/2$-phase-shifted Lorentzian functions $G(x) = \sum_{p=0}^{\infty} 2ig(x, (p + 0.5)\pi)$, where p is an index of summation, and $g(x, \theta) = (x + i\theta)^{-1}$ is a complex Lorentzian function. The real and imaginary parts of $ig(x, \theta)$ satisfy the Kramers–Kronig relation. This series has a broadened step-function-like imaginary part that is equal to the hyperbolic tangent function, $\tanh(x)$.

Because the imaginary part of the optical susceptibility must be an odd function due to causality, and it can be assumed to be zero above 10 eV for SWCNTs [8], we express $\tilde{\chi}_{\mathrm{C}}(\omega)$ as

$$\tilde{\chi}_{\mathrm{C}}(\omega) = A_{\mathrm{C}}\left[G\left((\omega - \omega_{\mathrm{C}})\gamma_{\mathrm{C}}^{-1}\right) + G\left((\omega + \omega_{\mathrm{C}})\gamma_{\mathrm{C}}^{-1}\right) + G((\omega - \omega_{\mathrm{cut}})\gamma_{\mathrm{cut}}^{-1}) + G((\omega + \omega_{\mathrm{cut}})\gamma_{\mathrm{cut}}^{-1})\right], \tag{S1}$$

which has the spectral shape displayed in Figure 4c. On the right-hand side, the first and second terms represent the rise of the continuum band (dark blue curve). In addition, A_{C} is the absorption strength, and ω_{c} and γ_{C}^{-1} are parameters specifying the center frequency and gradient at $\omega = \omega_{\mathrm{C}}$, respectively. The third and fourth terms represent the drop of the continuum band (light blue curve), and the cutoff energy of $\hbar\omega_{\mathrm{cut}}$ = 6 eV and the gradient of $(\hbar\gamma_{\mathrm{cut}})^{-1}$ = 1 eV^{-1} are assumed based on previous work [8]. While the imaginary part of this infinite series is exactly given by the sum of hyperbolic tangent functions, the real part has no simple expression, and we calculate the series over $p = 0,1,2, \ldots 100$ because it no longer changes substantially above $p \sim 100$. Here, we only consider contributions from the S_{11} continuum band for simplicity. This simplification is valid in the energy

range from the far-infrared to visible range around the S_{22} exciton resonance and is sufficient for applications in the near-infrared region.

Supplementary Note 3: The form of transfer and propagation transfer matrices

When light enters from media i to medium j, the transfer matrix ($\mathbf{M}_{ji}$) for normal incidence is given by

$$\mathbf{M}_{ji} = \frac{1}{2}\begin{pmatrix} 1+\tilde{n}_i/\tilde{n}_j & 1-\tilde{n}_i/\tilde{n}_j \\ 1-\tilde{n}_i/\tilde{n}_j & 1+\tilde{n}_i/\tilde{n}_j \end{pmatrix}. \quad \text{(S2).}$$

Here, $\tilde{n}_{i(j)}$ is the complex refractive index of the medium i (j). The propagation matrix ($\mathbf{\Phi}_i$) is given by

$$\mathbf{\Phi}_i = \begin{pmatrix} \exp(i2\pi\tilde{n}_i d/\lambda) & 0 \\ 0 & \exp(-i2\pi\tilde{n}_i d/\lambda) \end{pmatrix}, \quad \text{(S3)}$$

where λ is the wavelength in vacuum and d is the thickness of the medium.

Supplementary Note 4: Exciton oscillator strength of carbon nanotubes in membranes

The distribution of the orientations of SWCNTs in the membranes is different from that in the solution. In the solution, the tube axes of the SWCNTs are oriented in all directions with equal probability. Then, the measured absorption cross section of carbon atoms ($\sigma_{\mathrm{C}}(\omega)$) for excitons can be related to the oscillator strength along the tube axis ($f_{\parallel}$) by $f_{\parallel}/3 \propto \int \sigma_{\mathrm{C}}(\omega)d\omega$. Regarding the membrane, the transmission electron microscope observation revealed that SWCNTs exhibited nearly in-plane orientation, and few SWCNTs were oriented perpendicular to the membrane surface (not displayed here). This result is reasonable, given the fabrication method using vacuum filtration. The σ_{C} of the membrane is related to $f_{\parallel}$ as $f_{\parallel}/2 \propto \int \sigma_{\mathrm{C}}(\omega)d\omega$. Based on our observation of the reduction of $\int \sigma_{\mathrm{C}}(\omega)d\omega$ to ~50% displayed in the inset of Fig. 5 of the main text, the oscillator strength along the tube axis was reduced to 33% with the membrane fabrication, presumably because of the higher dielectric screening in the membrane due to bundling and/or unintentional carrier doping as inferred from the low energy response below ~0.3 eV shown in Fig. 5. In this estimation, we neglected the local field correction for the membrane, and a deeper discussion requires detailed investigation of the SWCNT morphologies in the membrane.

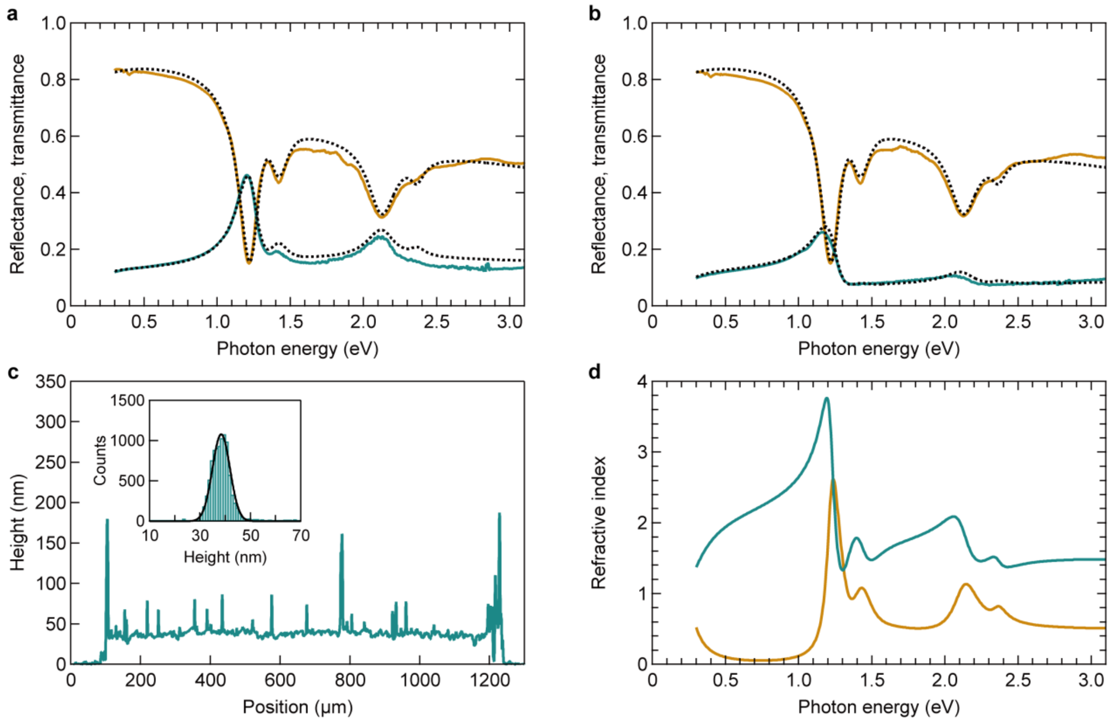

Supplementary Figure 2: Optical spectra and thickness profile of (6,5) carbon nanotube membrane. (a,b) Reflectance (green) and transmittance (orange) spectra of the on-sapphire membrane composed of (6,5) single-walled carbon nanotubes (SWCNTs). Probe light incident on the SWCNT (a) and sapphire (b) sides. The dotted curves represent the fitting results. (c) Height profile and height histogram (inset) of the SWCNT membrane on a silicon substrate. The black solid curve is the fitting result using the Gaussian function with an average thickness of 38 nm and a standard deviation of 4 nm. (d) Complex refractive index spectrum. The real and imaginary parts are indicated by green and orange, respectively.

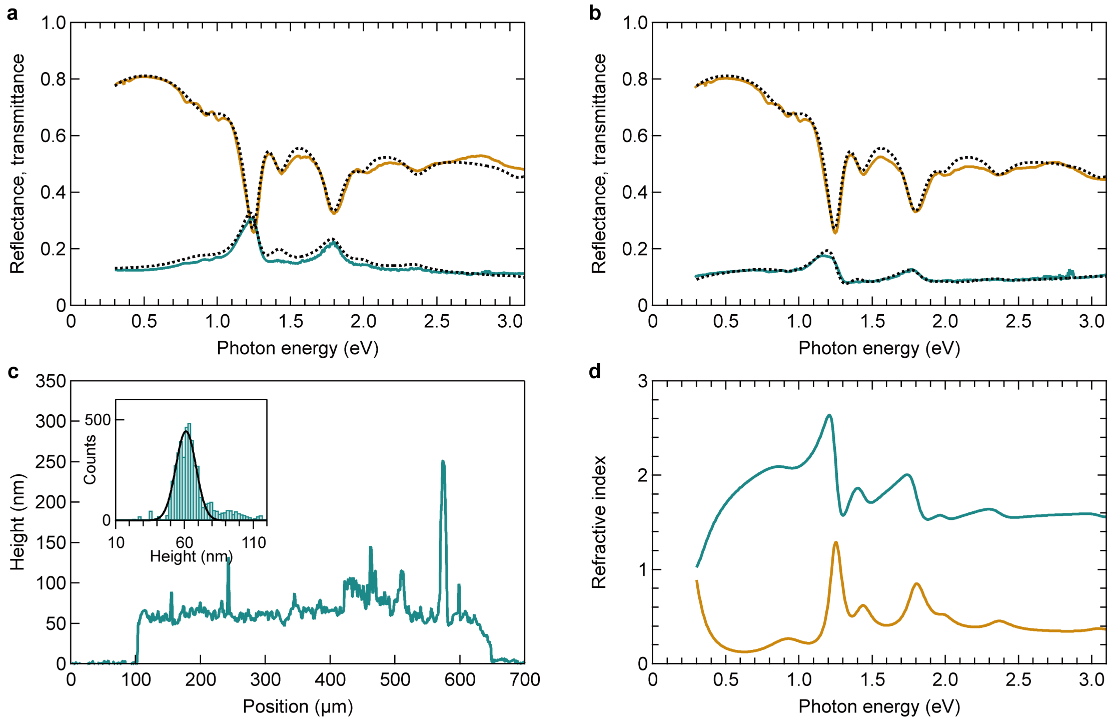

Supplementary Figure 3: Optical spectra and thickness profile of (8,3) carbon nanotube membrane. (a,b) Reflectance (green) and transmittance (orange) spectra of the on-sapphire membrane composed of (8,3) single-walled carbon nanotubes (SWCNTs); Probe light incident on the SWCNT (a) and sapphire (b) sides. The dotted curves represent the fitting results. (c) Height profile and height histogram (inset) of the SWCNT membrane on a silicon substrate. The black solid curve is the fitting result using the Gaussian function with an average thickness of 60 nm and a one standard deviation of 7 nm. (d) Complex refractive index spectrum. The real and imaginary parts are indicated by green and orange, respectively.

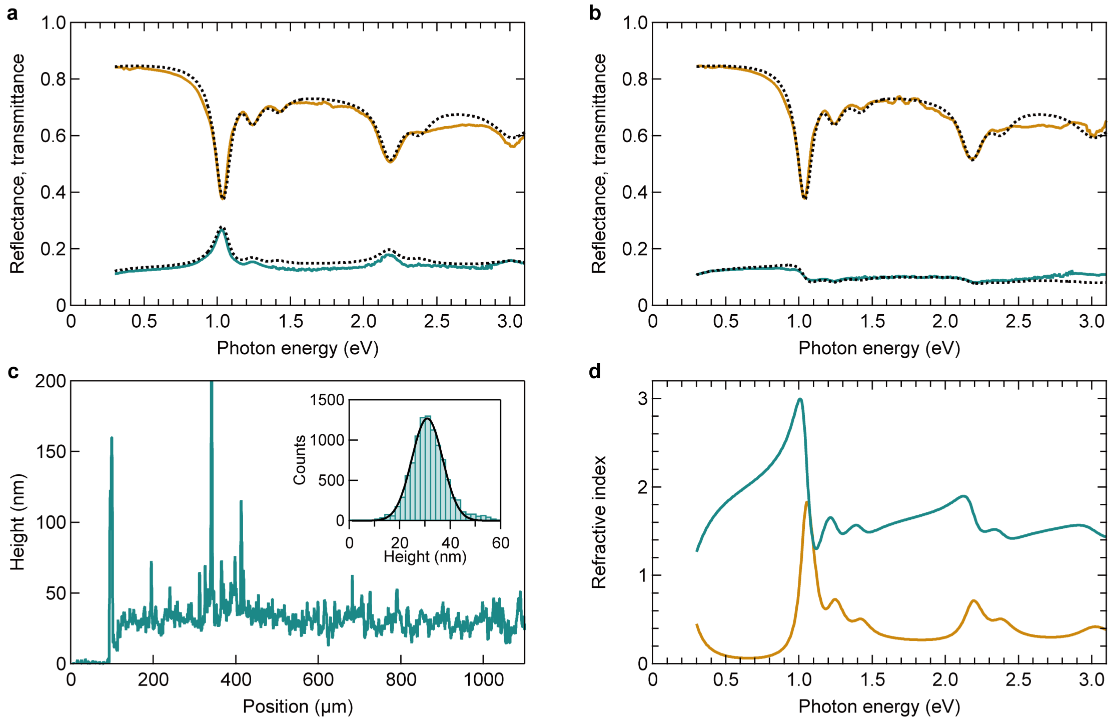

Supplementary Figure 4: Optical spectra and thickness profile of (9,2) carbon nanotube membrane. (a,b) Reflectance (green) and transmittance (orange) spectra of the on-sapphire membrane composed of (9,2) single-walled carbon nanotubes (SWCNTs); Probe light incident on the SWCNT (a) and sapphire (b) sides. The dotted curves represent the fitting results. (c) Height profile and height histogram (inset) of the SWCNT membrane on a silicon substrate. The black solid curve is the fitting result using the Gaussian function with an average thickness of 30 nm and a one standard deviation of 6 nm. (d) Complex refractive index spectrum. The real and imaginary parts are indicated by green and orange, respectively.

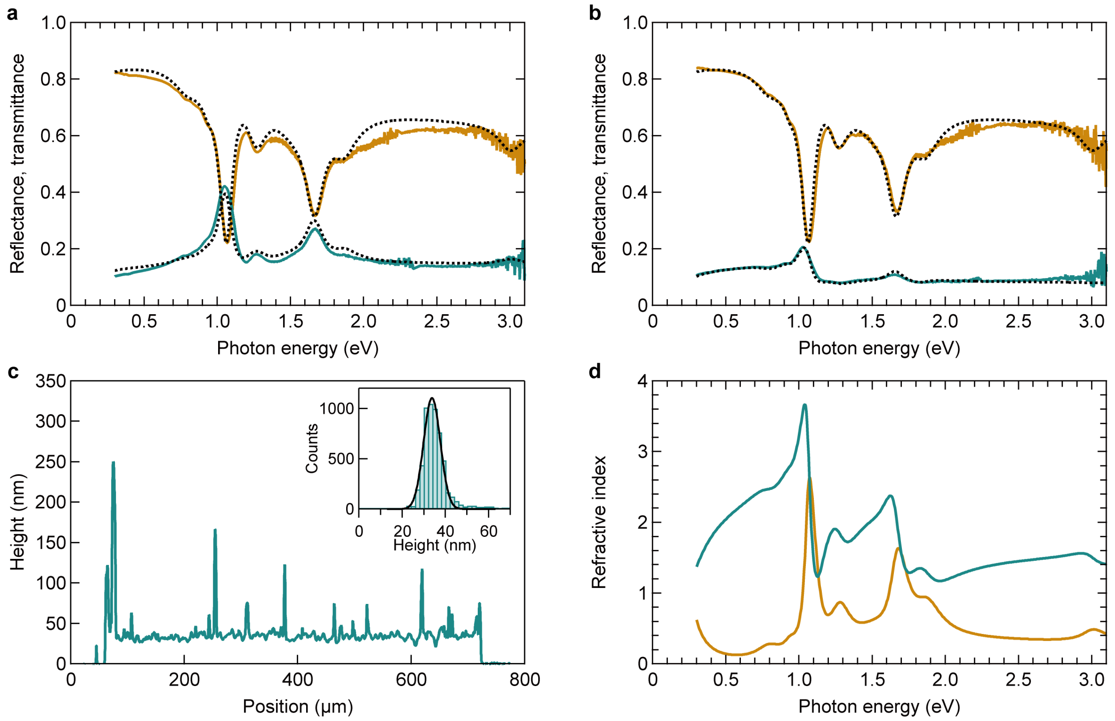

Supplementary Figure 5: Optical spectra and thickness profile of (9,4) carbon nanotube membrane. (a,b) Reflectance (green) and transmittance (orange) spectra of the on-sapphire membrane composed of (9,2) single-walled carbon nanotubes (SWCNTs); Probe light incident on the SWCNT (a) and sapphire (b) sides. The dotted curves represent the fitting results. (c) Height profile and height histogram (inset) of the SWCNT membrane on a silicon substrate. The black solid curve is the fitting result using the Gaussian function with an average thickness of 32 nm and a one standard deviation of 4 nm. (d) Complex refractive index spectrum. The real and imaginary parts are indicated by green and orange, respectively.

Supplementary Table 1: Oscillator parameters for the (10,3) single-walled carbon nanotube membrane. S_{jj} and P_{jj} denote the j-th subband exciton state and its phonon sideband originating from the longitudinal optical phonons, respectively.

Oscillators			
Lorentz type ($\tilde{\chi}_{\mathrm{L}}^{i}$)	f_{L}^{i} (g^{-1} cm^{3} eV^{2})	$\hbar\omega_{\mathrm{L}}^{i}$ (eV)	$\hbar\gamma_{\mathrm{L}}^{i}$ (eV)
S_{11} (P_{11})	1.00 (0.30)	0.94 (1.14)	0.09 (0.15)
S_{22} (P_{22})	0.54 (0.10)	1.89 (2.12)	0.11 (0.15)
S_{33}	0.71	2.87	0.25
Other chiralities	0.09, 0.12	0.80, 1.43	0.10, 0.16
Continuum energy band ($\tilde{\chi}_{\mathrm{c}}$)	A_{C} (g^{-1}cm^{3})	$\hbar\omega_{\mathrm{C}}$ (eV)	$\hbar\gamma_{\mathrm{C}}$ (eV)
	0.54	1.18	0.15
Drude response ($\tilde{\chi}_{\mathrm{D}}$)	A_{D} (g^{-1} cm^{3} eV^{2})	$\hbar\gamma_{\mathrm{D}}$ (eV)	
	0.24	0.13	
Background	χ_{B} (g^{-1} cm^{3})		
	2.25		

Supplementary Table 2: Oscillator parameters for the (6,5) single-walled carbon nanotube membrane. S_{jj} and P_{jj} denote the j-th subband exciton state and its phonon sideband originating from the longitudinal optical phonons, respectively.

Oscillators			
Lorentz type ($\tilde{\chi}_{\mathrm{L}}^{i}$)	f_{L}^{i} (g^{-1} cm^3 eV^2)	$\hbar\omega_{\mathrm{L}}^{i}$ (eV)	$\hbar\gamma_{\mathrm{L}}^{i}$ (eV)
S_{11} (P_{11})	1.50 (0.55)	1.22 (1.42)	0.08 (0.15)
S_{22} (P_{22})	1.01 (0.15)	2.12 (2.36)	0.18 (0.11)
Continuum energy band ($\tilde{\chi}_{\mathrm{c}}$)	A_{C} (g^{-1}cm^3)	$\hbar\omega_{\mathrm{C}}$ (eV)	$\hbar\gamma_{\mathrm{C}}$ (eV)
	0.74	1.47	0.15
Drude response ($\tilde{\chi}_{\mathrm{D}}$)	A_{D} (g^{-1} cm^3 eV^2)	$\hbar\gamma_{\mathrm{D}}$ (eV)	
	0.35	0.13	
Background	χ_{B} (g^{-1} cm^3)		
	2.69		

Supplementary Table 3: Oscillator parameters for the (8,3) single-walled carbon nanotube membrane. S_{jj} and P_{jj} denote the j-th subband exciton state and its phonon sideband originating from the longitudinal optical phonons, respectively.

Oscillators			
Lorentz type ($\tilde{\chi}_{\mathrm{L}}^{i}$)	f_{L}^{i} (g^{-1} cm^{3} eV^{2})	$\hbar\omega_{\mathrm{L}}^{i}$ (eV)	$\hbar\gamma_{\mathrm{L}}^{i}$ (eV)
S_{11} (P_{11})	0.59 (0.30)	1.24 (1.43)	0.09 (0.15)
S_{22} (P_{22})	0.48 (0.08)	1.79 (1.99)	0.14 (0.13)
S_{33}	0.06	3.04	0.17
Other chiralities	0.28, 0.16	0.93, 2.36	0.34, 0.19
Continuum energy band ($\tilde{\chi}_{\mathrm{c}}$)	A_{C} (g^{-1}cm^{3})	$\hbar\omega_{\mathrm{C}}$ (eV)	$\hbar\gamma_{\mathrm{C}}$ (eV)
	0.52	1.47	0.15
Drude response ($\tilde{\chi}_{\mathrm{D}}$)	A_{D} (g^{-1} cm^{3} eV^{2})	$\hbar\gamma_{\mathrm{D}}$ (eV)	
	0.44	0.13	
Background	χ_{B} (g^{-1} cm^{3})		
	2.52		

Supplementary Table 4: Oscillator parameters for the (9,2) single-walled carbon nanotube membrane. S_{jj} and P_{jj} denote the j-th subband exciton state and its phonon sideband originating from the longitudinal optical phonons, respectively.

Oscillators			
Lorentz type ($\tilde{\chi}_{\mathrm{L}}^{i}$)	f_{L}^{i} (g^{-1} cm^{3} eV^{2})	$\hbar\omega_{\mathrm{L}}^{i}$ (eV)	$\hbar\gamma_{\mathrm{L}}^{i}$ (eV)
S_{11} (P_{11})	0.81 (0.26)	1.04 (1.24)	0.09 (0.14)
S_{22} (P_{22})	0.52 (0.21)	2.18 (2.38)	0.15 (0.17)
S_{33}	0.39	3.01	0.27
Other chiralities	0.11	1.42	0.14
Continuum energy band ($\tilde{\chi}_{\mathrm{c}}$)	A_{C} (g^{-1}cm^{3})	$\hbar\omega_{\mathrm{C}}$ (eV)	$\hbar\gamma_{\mathrm{C}}$ (eV)
	0.38	1.26	0.15
Drude response ($\tilde{\chi}_{\mathrm{D}}$)	A_{D} (g^{-1} cm^{3} eV^{2})	$\hbar\gamma_{\mathrm{D}}$ (eV)	
	0.29	0.12	
Background	χ_{B} (g^{-1} cm^{3})		
	2.10		

Supplementary Table 5: Oscillator parameters for the (9,4) single-walled carbon nanotube membrane. S_{jj} and P_{jj} denote the j-th subband exciton state and its phonon sideband originating from the longitudinal optical phonons, respectively.

Oscillators			
Lorentz type ($\tilde{\chi}_\mathrm{L}^i$)	f_L^i (g^{-1} cm^3 eV^2)	$\hbar\omega_\mathrm{L}^i$ (eV)	$\hbar\gamma_\mathrm{L}^i$ (eV)
S_{11} (P_{11})	0.94 (0.44)	1.06 (1.27)	0.06 (0.16)
S_{22} (P_{22})	0.91 (0.36)	1.66 (1.86)	0.11 (0.19)
S_{33}	0.28	3.00	0.19
Other chiralities	0.15, 0.08, 0.30	0.80, 0.94, 1.52	0.21, 0.10, 0.28
Continuum energy band ($\tilde{\chi}_\mathrm{c}$)	A_C (g^{-1}cm^3)	$\hbar\omega_\mathrm{C}$ (eV)	$\hbar\gamma_\mathrm{C}$ (eV)
	0.47	1.30	0.15
Drude response ($\tilde{\chi}_\mathrm{D}$)	A_D (g^{-1} cm^3 eV^2)	$\hbar\gamma_\mathrm{D}$ (eV)	
	0.40	0.13	
Background	χ_B (g^{-1} cm^3)		
	2.31		

Supplementary Table 6: Empirical parameters for the complex refractive index spectrum of a single-chirality semiconducting single-walled carbon nanotube (SWCNT) membrane. S_{11}^* and S_{22}^* denote the first and second subband exciton energies of an individual SWCNT reported [9], respectively. Angle brackets indicate an average value.

Oscillators			
Lorentz type ($\tilde{\chi}_{\mathrm{L}}^{i}$)	$\langle f_{\mathrm{L}}^{i} \rangle$ (g^{-1} cm^3 eV^2)	$\hbar\omega_{\mathrm{L}}^{i}$ (eV)	$\hbar\langle \gamma_{\mathrm{L}}^{i} \rangle$ (eV)
S_{11} exciton (phonon sideband)	0.97 (0.37)	S_{11}*−0.08 (S_{11}*+0.12)	0.08 (0.15)
S_{22} exciton (phonon sideband)	0.69 (0.18)	S_{22}*−0.08 (S_{22}*+0.13)	0.14 (0.15)
Continuum energy band ($\tilde{\chi}_{\mathrm{c}}$)	$\langle A_{\mathrm{C}} \rangle$ ($g^{-1}cm^3$)	$\hbar\omega_{\mathrm{C}}$ (eV)	$\hbar\langle \gamma_{\mathrm{C}} \rangle$ (eV)
	0.53	S_{11}*+0.16	0.15
Drude response ($\tilde{\chi}_{\mathrm{D}}$)	$\langle A_{\mathrm{D}} \rangle$ (g^{-1} cm^3 eV^2)	$\hbar\langle \gamma_{\mathrm{D}} \rangle$ (eV)	
	0.34	0.13	
Background	$\langle \chi_{\mathrm{B}} \rangle$ (g^{-1} cm^3)		
	2.37		

References

[1] X. He, W. Gao, L. Xie, B. Li, Q. Zhang, S. Lei, J. M. Robinson, E. H. Haroz, S. K. Doorn, W. Wang, R. Vajtai, P. M. Ajayan, W. W. Adams, R. H. Hauge, and J. Kono, "Wafer-scale monodomain films of spontaneously aligned single-walled carbon nanotubes," *Nat. Nanotechnol.*, vol. 11, pp. 633–638, 2016.

[2] Y. Yomogida, T. Tanaka, M. Zhang, M. Yudasaka, X. Wei, and H. Kataura, "Industrial-scale separation of high-purity single-chirality single-wall carbon nanotubes for biological imaging," *Nat. Commun.*, vol. 7, p. 12056, 2016.

[3] Y. Yomogida, T. Tanaka, M. Tsuzuki, X. Wei, and H. Kataura, "Automatic sorting of single-chirality single-wall carbon nanotubes using hydrophobic cholates: implications for multicolor near-infrared optical technologies," *ACS Appl. Nano Mater.*, vol. 3, pp.11289–11297, 2020.

[4] M.-F. Yu, O. Lourie, M. J. Dyer, K. Moloni, T. F. Kelly, and R. S. Ruoff, "Strength and breaking mechanism of multiwalled carbon nanotubes under tensile load," *Science*, vol. 287, pp. 637–640, 2000.

[5] K. Liu, X. Hong, S. Choi, C. Jin, R. B. Capaz, J. Kim, W. Wang, X. Bai, S. G. Louie, E. Wang, and F. Wang, "Systematic determination of absolute absorption cross-section of individual carbon nanotubes," *Proc. Natl. Acad. Sci. USA*, vol. 111, pp. 7564–7569, 2014.

[6] Y. Miyauchi, M. Oba, and S. Maruyama, "Cross-polarized optical absorption of single-walled nanotubes by polarized photoluminescence excitation spectroscopy," *Phys. Rev. B*, vol. 74, p. 205440, 2006.

[7] F. Katsutani, W. Gao, X. Li, Y. Ichinose, Y. Yomogida, K. Yanagi, and J. Kono, "Direct observation of cross-polarized excitons in aligned single-chirality single-wall carbon nanotubes," *Phys. Rev. B*, vol. 99, p. 035426, 2019.

[8] R. Senga, T. Pichler, Y. Yomogida, T. Tanaka, H. Kataura, and K. Suenaga, "Direct proof of a defect-modulated gap transition in semiconducting nanotubes," *Nano Lett.*, vol. 18, pp. 3920–3925, 2018.

[9] K. Liu, J. Deslippe, F. Xiao, R. B. Capaz, X. Hong, S. Aloni, A. Zettl, W. Wang, X. Bai, S. G. Louie, E. Wang, and F. Wang. "An atlas of carbon nanotube optical transitions," *Nat. Nanotechnol.*, vol. 7, pp. 325–329, 2012.
